# Integration of Conventional and Virtual Reality Approaches in Augmented Reality for Theory-Based Psychoeducational Intervention Design for Chronic Low Back Pain: Scoping Review

**DOI:** 10.2196/59611

**Published:** 2025-01-20

**Authors:** Robin Conen, Steffen Mueller, Ana Nanette Tibubos

**Affiliations:** 1 Department of Nursing Science, Diagnostics in Healthcare and eHealth Trier University Trier Germany; 2 Department of Computer Science/Therapeutic Science Trier University of Applied Sciences Trier Germany; 3 Department of Psychosomatic Medicine and Psychotherapy University Medical Center of the Johannes Gutenberg-University Mainz Mainz Germany

**Keywords:** augmented reality, virtual reality, chronic low back pain, education, pain management, intervention

## Abstract

**Background:**

Psychoeducation positively influences the psychological components of chronic low back pain (CLBP) in conventional treatments. The digitalization of health care has led to the discussion of virtual reality (VR) interventions. However, CLBP treatments in VR have some limitations due to full immersion. In comparison, augmented reality (AR) supplements the real world with virtual elements involving one’s own body sensory perception and can combine conventional and VR approaches.

**Objective:**

The aim of this study was to review the state of research on the treatment of CLBP through psychoeducation, including immersive technologies, and to formulate suggestions for psychoeducation in AR for CLBP.

**Methods:**

A scoping review following PRISMA (Preferred Reporting Items for Systematic Reviews and Meta-Analyses) guidelines was performed in August 2024 by using Livivo ZB MED, PubMed, Web of Science, American Psychological Association PsycINFO (PsycArticle), and PsyArXiv Preprints databases. A qualitative content analysis of the included studies was conducted based on 4 deductively extracted categories.

**Results:**

We included 12 studies published between 2019 and 2024 referring to conventional and VR-based psychoeducation for CLBP treatment, but no study referred to AR. In these studies, educational programs were combined with physiotherapy, encompassing content on pain biology, psychological education, coping strategies, and relaxation techniques. The key outcomes were pain intensity, kinesiophobia, pain catastrophizing, degree of disability, quality of life, well-being, self-efficacy, depression, attrition rate, and user experience. Passive, active, and gamified strategies were used to promote intrinsic motivation from a psychological point of view. Regarding user experience from a software development perspective, user friendliness, operational support, and application challenges were recommended.

**Conclusions:**

For the development of a framework for an AR-based psychoeducational intervention for CLBP, the combination of theories of acceptance and use of technologies with insights from health psychological behavior change theories appears to be of great importance. An example of a theory-based design of a psychoeducation intervention in AR for CLBP is proposed and discussed.

## Introduction

Globally, 60%-80% of adults experience low back pain, with 10% developing chronic forms, of which 85% are classified as chronic nonspecific low back pain without a clear etiology [1]. Owing to the limited efficacy and adverse effects of pharmacological approaches, there is a need for nonpharmacological alternatives [2] to improve treatment outcomes [3] and develop effective behavioral interventions [4]. Treatment guidelines recommend behavioral modification, exercise, psychoeducation [5-7], and physiotherapy for trunk muscle strengthening [8-11] to reduce pain and disability.

Educational interventions for chronic low back pain (CLBP) provide knowledge about the condition, coping strategies, and physical activity [3,12], with the objective of enhancing the quality of life and symptom management by mitigating anxiety, kinesiophobia, hyperactive pain behavior, and depression, which are risk factors for pain chronification. Additionally, psychoeducation fosters self-efficacy to break the cycle between anxiety and pain [13,14].

Many traditional interventions to boost physical activity, which is key for CLBP treatment, rely on intention theories for modifying health behavior [15]. A prominent intention theory is the Unified Theory of Acceptance and Use of Technology 2 (UTAUT 2) by Venkatesh et al [16], which examines the acceptance and use of technologies. It has gained recognition in fields such as education, e-commerce, and health research with advancing health technology [17,18]. UTAUT 2 explains the formation of intention for technology use through the constructs of performance expectancy, effort expectancy, social influence, facilitating conditions, hedonic motivation, price value, and habit. These factors may also be useful for predicting the intentions of patients with CLBP toward educational technology adoption. Furthermore, insights from theories focusing on health behavior change may prove fruitful to consider when attempting to change health behavior through the use of a new technology. For instance, Schwarzer’s Health Action Process Approach model [19], commonly used in health behavior interventions, highlights self-efficacy and outcome expectation. The Health Action Process Approach distinguishes between intention formation and implementation as well as between nonintenders, intenders, and actors, each requiring tailored interventions to promote self-efficacy, information, and support in implementation [20]. Another example is Michie’s Behavior Change Technique (BCT) taxonomy with 93 BCTs outlining strategies for successful behavior change [21].

Immersive technologies can be characterized on the Reality-Virtuality Continuum by Milgram and Kishino [22]. They demonstrate visual display technologies ranging from real to virtual environments, including augmented reality (AR) and virtual reality (VR) [23-25]. AR enables the concurrent presence and interaction of digital and physical elements within real-world environments in real time. VR, in contrast, enables complete immersion in VR and represents the extreme of Milgram’s continuum between reality and virtuality [26].

With regard to research in immersive technologies such as VR in the treatment of CLBP, VR-based treatments turned out to be promising in reducing acute, experimental, and chronic pain and can complement conventional CLBP treatments [27].

VR has proven effective in treating acute pain [24] by redirecting attention from unpleasant stimuli such as back pain to more pleasant visual, auditory, and tactile stimuli [27]. VR interventions were found to reduce pain intensity, catastrophizing symptoms, and psychological symptoms in patients with CLBP after one session through distraction, indicating the direct influence of VR on pain perception [26]. Other VR studies demonstrated the feasibility and efficacy of VR for CLBP as an alternative approach, such as VR applications with graded exposure during walking and grasping with integrated game design [28], self-administered VR therapy for CLBP at home [29], and its implementation even during COVID-19 [30]. A recent meta-analysis also showed that kinesiophobia and pain intensity in CLBP can be reduced through VR training [31].

Although there is some evidence for the safety and tolerability of VR treatment for CLBP, most studies lack methodological quality and results were limited to short-term effects. Studies on safety, acceptance, and satisfaction are lacking, including targeted investigations of the risks of spinal pain caused by VR [32]. Thus, while VR is promising in reducing CLBP symptoms, AR might offer additional benefits through the integration of physical and virtual elements, thereby reducing VR-associated discomfort. AR enables the coexistence and interaction of virtual and physical objects in real time in the real world, thus combining the advantages of VR while mitigating its limitations such as cybersickness and visual discomfort [33]. AR can enhance interaction, presence, intuitiveness, and pedagogical flexibility by enriching the real world with digital information, accommodating various learning styles, and facilitating teaching and learning [34]. Despite these presumed advantages of AR, to our knowledge, there are no empirical studies of AR-based treatment for CLBP.

In summary, pain treatment guidelines emphasize the key role of educational CLBP treatment to counteract psychological chronification and promote self-efficacy according to health behavior change models. Furthermore, when health behavior change is addressed using a new technology, a joint consideration of health psychological models with theories of acceptance from a technological perspective, like the UTAUT 2 [35], is considered useful for successful implementation. Existing studies with immersive technology [24,26-31] demonstrated positive effects for CLBP treatment in VR incorporating psychoeducational elements. However, these VR studies have methodological shortcomings and gaps regarding dimensions of user experience such as satisfaction and acceptance. Therefore, by formulating research questions using the PICO (Population, Intervention, Comparison, Outcome) framework, this scoping review aims to first examine research in patients with CLBP (P) receiving psychoeducation through immersive technology (I) compared to conventional psychoeducation (C) to improve pain relief and pain-psychological variables (O) and second on the basis of the results of the literature analysis, to develop an intervention design for AR-based psychoeducation in patients with CLBP that combines conventional methods with immersive technology based on a technology acceptance model to promote acceptance and pain management.

## Methods

We investigated the research question through a scoping review and followed the PRISMA (Preferred Reporting Items for Systematic Reviews and Meta-Analyses) extension for scoping reviews [36]. This review includes studies that used psychoeducation for CLBP and chronic pain treatment: (1) conventionally, (2) with immersive technology in VR or AR, or (3) a combination of both, conventional therapy with VR or AR technology use. Only papers published in English or German in 2019-2024 were considered, wherein clinical guidelines were generally updated every 3-5 years with new evidence [37]. The exclusion criteria were as follows: (1) psychiatric patients, (2) acute back pain, (3) back pain after medical procedures, and (4) other specific pain conditions and pharmacological interventions. Scientific investigations or studies in journals or textbooks were included, regardless of the scientific methodology used. An electronic search was performed in August 2024 by using predefined English terms: (“chronic low back pain” OR “CLBP” OR “chronic pain”) AND ((“virtual reality” OR “augmented reality”) OR (“education” OR “multimodal pain therapy” OR “psychological intervention”)). Reviewer RC used Citavi to search for in vivo ZB MED and PubMed, and a manual search was conducted in the Web of Science, American Psychological Association PsycINFO, and PsyArXiv Preprints. The search was conducted in line with the Joanna Briggs Institute methodology for scoping reviews, extending the PRISMA statement [38]. In accordance with Arksey and O’Malley’s [39] recommendations for scoping reviews, we did not include a formal quality assessment of the incorporated research. The selection process was initially based on a review of titles and abstracts regarding the inclusion and exclusion criteria, followed by an assessment of the full text by a reviewer (RC) and double-checked by another reviewer (ANT). Both reviewers (RC and ANT) extracted the following information from the included studies by using Microsoft Excel, following the Joanna Briggs Institute model: (1) citation, (2) context, (3) participant characteristics, (4) study aim, (5) methodology, (6) results, (7) interventions, (8) limitations, (9) key results related to review questions, and (10) future research areas [40]. Data analysis by the first reviewer (RC) utilized a qualitative content analysis [41]. A deductive approach was used to extract relevant categories for achieving the research objective. Guidelines for the treatment of CLBP [42] as well as recommendations of the World Health Organization for digital health interventions [43] served as the basis for this. Subsequently, 4 categories were extracted to capture all the essential aspects relevant to the design of the envisaged intervention. The categories are as follows: (1) content of CLBP-specific education, (2) factors based on the psychology of learning for the intervention design, (3) technical conditions (framework) for CLBP interventions, and (4) outcome measures of the educational interventions for CLBP.

## Results

### Study Selection

**Figure 1:**
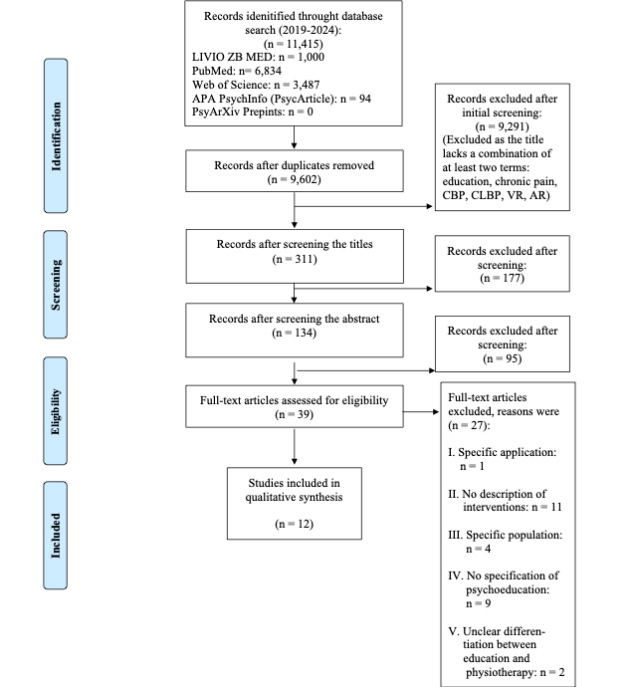
PRISMA (Preferred Reporting Items for Systematic Reviews and Meta-Analyses) flow diagram of the study selection process. APA: American Psychological Association; AR: augmented reality; CBP: chronic back pain; CLBP: chronic low back pain; VR: virtual reality.

The study selection process, as shown in Figure 1, began with a database search that yielded 11,415 results. A total of 9602 titles were screened for the following terms: education, chronic pain, chronic back pain, CLBP, VR, and AR. The title should contain a minimum of 2 of the following keywords: education, chronic pain, chronic back pain, CLBP, VR, or AR; 9291 papers were excluded due to the lack of appearance of at least 2 of the defined terms. After applying the inclusion and exclusion criteria to 311 publications, 177 studies were excluded based on the titles and 95 were excluded based on the abstracts. After reviewing the full texts of the remaining 39 publications, 27 were excluded for (1) specific applications (eg, doctor-patient communication), (2) insufficient intervention descriptions, (3) overly specific populations (eg, elite athletes, primary school students, nursing staff), (4) unspecified psychoeducation (eg, cognitive behavioral therapy [CBT], cognitive functional training), and (5) unclear differentiation between education and physiotherapy in intervention design. Finally, the scoping review analyzed 12 publications, displayed in Table 1 [44-55].

The studies originated from Italy, Spain, the Netherlands, France, Chile, India, and Tunisia (8.33% each), Germany (25%), and the United States (16.67%). The review included 9 empirical studies (1 interview study, 8 interventional studies) and 3 reviews (1 systematic, 1 scoping, and 1 narrative review). All studies were peer-reviewed, except the narrative review. The results of the analysis of the 12 publications included are presented below according to the 4 defined categories.

**Table 1:** Included studies applying psychoeducation by using conventional approaches and virtual reality approaches for the treatment of chronic low back pain [44-55].

Author	Study title	Journal name
Salazar-Méndez et al [44], 2024	Pain Neuroscience Education for Patients With Chronic Pain: A Scoping Review From Teaching-Leaning Strategies, Educational Level, and Cultural Perspective	Patient Education and Counseling
Ferlito et al [45], 2022	Pain Education in the Management of Patients with Chronic Low Back Pain: A Systematic Review	Journal of Functional Morphology and Kinesiology
Rim et al [46], 2022	Efficiency of Associating Therapeutic Patient Education with Rehabilitation in the Management of Chronic Low Back Pain: A Randomized Controlled Trial	Korean Journal of Family Medicine
Sidiq et al [47], 2024	Effects of Pain Education on Disability, Pain, Quality of Life, and Self-Efficacy in Chronic Low Back Pain: A Randomized Controlled Trial	PLOS One
Tomás-Rodríguez et al [48], 2024	Short- and Medium-Term Effects of a Single Session of Pain Neuroscience Education on Pain and Psychological Factors in Patients With Chronic Low Back Pain: A Single-Blind Randomized Clinical Trial	European Journal of Pain
Janik et al [49], 2024	Middle-Term Effects of Education Program in Chronic Low Back Pain Patients to an Adherence to Physical Activity: A Randomized Controlled Trial	Patient Education and Counseling
Lindner et al [50], 2020	Use of Virtual Reality as a Component of Acute and Chronic Pain Treatment	Anasthesiologie Intensivmedizin Notfallmedizin Schmerztherapie
Stamm et al [51], 2020	Virtual Reality in Pain Therapy: a Requirements Analysis for Older Adults With Chronic Back Pain	Journal of NeuroEngineering and Rehabilitation
Stamm et al [52], 2022	Virtual Reality Exergame for Supplementing Multimodal Pain Therapy in Older Adults With Chronic Back Pain	Virtual Reality
Brown et al [53], 2023	Chronic Pain Education Delivered With a Virtual Reality Headset in Outpatient Physical Therapy Clinics: A Multisite Exploratory Trial	American Journal of Translational Research
McConnell et al [54], 2024	A Multicenter Feasibility Randomized Controlled Trial Using a Virtual Reality Application of Pain Neuroscience Education for Adults With Chronic Low Back Pain	Annals of Medicine
de Vries et al [55], 2023	Pain Education and Pain Management Skills in Virtual Reality in the Treatment of Chronic Low Back Pain: A Multiple Baseline Single-Case Experimental Design	Behavior Research and Therapy

### Contents of CLBP-Specific Education

The systematic review evaluated clinical studies from 2011 to 2021 comparing pain education/CBT with conventional physiotherapy for CLBP [45]. Thirteen studies, including 12 randomized controlled trials with 1642 participants, were analyzed. Six studies demonstrated a significant reduction in pain compared with the control group. The review concluded that due to the multimodality and heterogeneity of treatments, no definitive statement can be made regarding the efficacy of pain education or CBT in patients with CLBP [45]. Seven studies included an educational program in conjunction with physiotherapy [45-48,52-54]. Educational content varied considerably, ranging from exclusive focus on pain biology [46-49,52,53,55] to the inclusion of psychological aspects [46,52-55] and multidisciplinary approaches [49]. Most of the programs [48,55] incorporated education on pain physiology, frequently based on the book “Explain Pain” [56] by Butler and Moseley. Psychological education encompassed topics such as physical activity [45,46,49,53], fear of physical activity, emotional management [45-49,51], lifestyle modifications, daily exercises [45,49,52], pain-specific coping strategies [45,52,54], pain sensitization [47,54,55], and relaxation techniques, including stress management and mindfulness [45,49,52-55] in 6 studies, of which 5 included VR interventions. The content, duration, and physiotherapeutic integration of the individual education programs can be found in Multimedia Appendix 1.

### Factors Based on Psychology of Learning for Intervention Design

This category encompasses factors of psychology of learning that are pertinent to the design of interactive interventions. Analysis of 3 studies showed that VR-based education employs passive mediation strategies such as informational videos and lectures (provided conventionally and in VR), alongside active and interactive strategies [44,50,54]. Three studies mentioned VR-based gamified approaches [50,52,55] and 2 studies [50,51] mentioned the promotion of intrinsic motivation.

#### Mediation Strategies

The included systematic review [44] examined the programs, cultural adaptations, and the efficacy of pain neuroscience education for chronic musculoskeletal pain, analyzing 71 studies that met our inclusion criteria and featured pain duration exceeding 3 months in adults. The analyzed studies explored pain neuroscience education in different settings by using various experimental designs, including secondary analyses of randomized controlled trials, and showed positive effects on pain and psychological variables. Despite cultural influences on pain-relevant factors, only 2 (3%) of the 71 studies culturally adapted the pain neuroscience education material. Passive teaching-learning strategies tended to yield better outcomes for pain and functionality, whereas active methods resulted in significant knowledge improvements, albeit with insufficient description. The outcomes of multimodal therapies for chronic pain depend on the individualized integration of pain-specific education, considering biopsychosocial factors, educational level, culture, and diverse learning methods and materials for conveying pain neuroscience content [44]. Interaction content is passively conveyed through videos or lectures [46-49,53], particularly VR-based 360° nature videos [53].

#### Gamification and Motivation Enhancement

The Pain-Neuro-Education 2.0 software utilized a VR headset with immersive footage and computer-generated images for visually and emotionally engaging educational and relaxation training for chronic pain. This included interactive emotion regulation exercises such as breathing and mindfulness exercises in natural environments [54]. The VR program Recupt was also used to convey information in an engaging manner by having the user shoot at the pain stimulus with a laser gun, among other things. In the spinal cord phase, participants focus on visual “pain gates” and breathing to metaphorically “close” them and experience relaxation-induced pain relief. The brain component elucidates the reduction in pain response through the visualization and reactivation of illuminated connections. The alarm center gameplay demonstrates how emotions, cognitions, and behaviors influence pain perception. Finally, participants envision the alarm center as a brain region that regulates pain stimuli in an aircraft cockpit [55]. The VR program ViRST provides a therapeutic, interactive user interface with task-based activities in a farm environment [52]. Patients visualize movements and exertion levels by using game-based biofeedback with progress tracking and narrative elements [51]. Exergames incorporate biofeedback such as heart rate variability via photoplethysmography to prevent overexertion in interactive scenarios [52]. Gamification can motivate and enhance therapy adherence by fulfilling the psychological needs of competence, autonomy, and relatedness through interactive knowledge transfer. It also improves user skills through playful activities [50]. In the long term, feedback should be framed positively to maintain intrinsic motivation [52]. Avatars manipulate body perception for therapeutic effects, with the Proteus effect causing users to adopt their avatar’s behavior in real life. Personalized avatars can amplify pain relief [50].

### Technical Conditions (Framework) for CLBP Intervention

The technical parameters of 3 enclosed VR studies, comprising 1 needs analysis [52] and 2 feasibility studies [52,53], provide insights into the design of AR-based education and identify potential areas of focus such as user-friendliness [51,53], operational support [51-53], and various application challenges [51,53]. The needs analysis was based on semistructured interviews (n=10) in focus groups to determine the requirements of older patients with chronic back pain, physiotherapists, and psychotherapists regarding VR pain therapy in terms of overall system, hardware, and software [51]. Findings emphasize that the designed system must be user-friendly; provide personalized instructions, demonstration videos, and individual guidance; and be available for rent. Assistants should support this system. Automatic breaks were considered crucial to avoid overexertion and pain aggravation. Activity should be limited to 30 minutes followed by a 15-minute rest. The study also highlighted the importance of balancing active therapy and relaxation. For hardware, it was determined that the VR headset must be independent and removable. Software design should consider user-friendliness by integrating the game environment with the level in-game environment for individual calibration of movement restrictions, particularly in gaming activities. Finally, a spacious room and wireless head-mounted display were considered essential for safety to prevent falls.

One feasibility study also emphasized the importance of safety aspects for the usability of VR headsets. The authors indicate that 93% of the application issues were associated with handling spatial and temporal limitations [53]. The second feasibility study demonstrated that disregarding body height (insufficient arm span) was perceived as disruptive [52]. Operational support software allows therapists to intervene during instances of pain, anxiety, or improper exercise execution by using a help button or emergency assistance [51]. Incorrect exercise execution is considered disruptive [52] and often lacks adequate support personnel for error correction or clinical assistance [53].

### Outcome Measures of Educational Interventions for CLBP

Of the 12 studies [44-55] reviewed, 8 [46-49,54,55] were quantitative interventional studies. Commonly evaluated outcomes in CLBP studies encompassed pain intensity [45-49,51,52,54,55], kinesiophobia [46,48,51-53,55], pain catastrophizing [48,53-55], disability [45-47,51,52,54], health-related quality of life [51,52,54], well-being [47], self-efficacy [47,54], depression [46,53], attrition rate [49,53], and VR intervention user experience [52,53]. A comprehensive table displaying the different methods used, study results, and conclusions for each of the included studies can be found in Multimedia Appendix 2. A list of the pain-specific constructs and their measurement tools assessed in the different studies can be found in Multimedia Appendix 3.

## Discussion

### Principal Findings

Psychoeducation is a key element for CLBP treatment. Psychoeducation provided in AR could offer more benefits than that in VR or with conventional methods by integrating physical and virtual elements. Additionally, psychoeducation in AR was assumed to be superior to VR due to reported VR-associated discomforts in CLBP treatment, such as cybersickness and visual discomfort. Therefore, we conducted a literature review in the first step to evaluate research on CLBP treatment through psychoeducation using conventional methods and immersive technologies in order to design a psychoeducational intervention in AR for CLBP. In the second step, we applied the extracted results of the literature review to a theoretical framework, in particular, the UTAUT 2, to provide a design example for AR-based psychoeducation for CLBP.

Our findings indicate that various educational programs were combined with physiotherapy [45-48,52-54]. These studies referred to conventional methods or VR-based interventions. No relevant study with AR for CLBP treatment was found. The varying educational content encompassed pain biology [46-49], psychological education on physical activity [45,46,49,53], anxiety management [45-49,51], lifestyle modifications, daily exercises [45,49,52], coping strategies [45,52,54], pain sensitization [47,54,55], and relaxation techniques [45,49,52-55]. Passive, active [44,50,54], and gamified strategies [50,52,55] were employed alongside the promotion of intrinsic motivation [50,51]. User-friendliness [51,53], operational support [51,52,54], and application challenges [52,53] were considered important for software development. The key variables of educational CLBP interventions included physiological variables such as pain intensity [45-49,51,52,54,55] and disability level [45-47,51,52,54]; psychological variables such as kinesiophobia [46,48,51,52,54,55], pain catastrophizing [48,53-55], quality of life [51,52,55], well-being [47], self-efficacy [47,54], and depression [46,53]; and technical variables such as dropout rates [49,53] and user experience [52,53].

**Figure 2:**
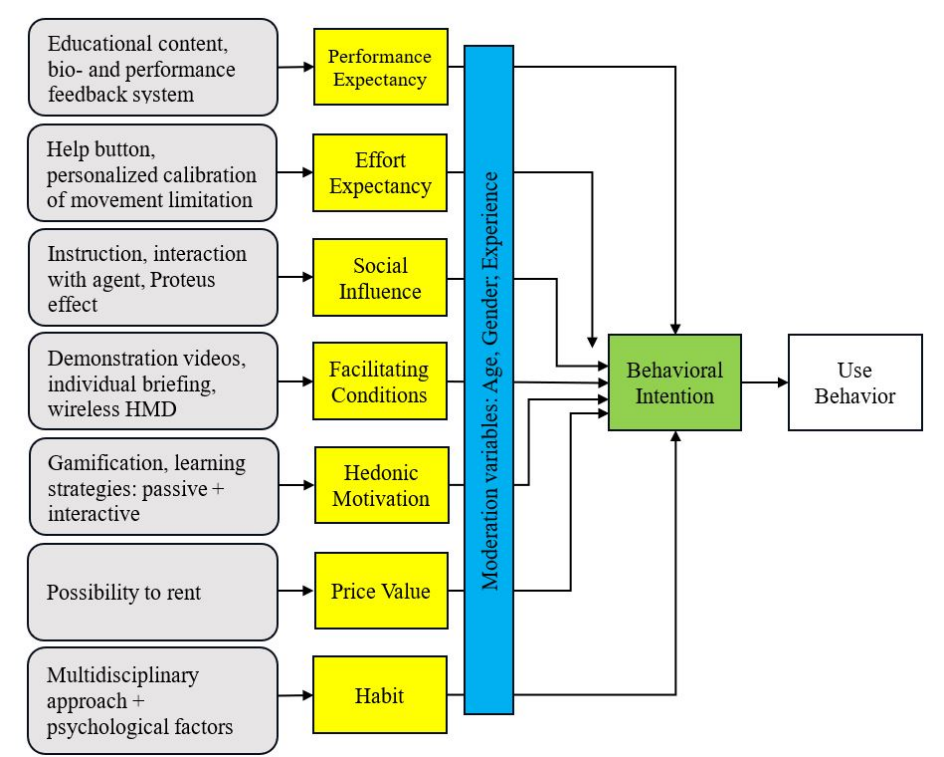
Exemplary mapping of the extracted findings from the literature review (grey) applied to the Unified Theory of Acceptance and Use of Technology to design an artificial reality–based psychoeducation for chronic low back pain. HMD: head-mounted display.

Our results elucidate key aspects of a useful design of a psychoeducational treatment in AR for CLBP, which does not exist to date, to the best of our knowledge. Our findings point out the relevance of the interplay of technical and psychological components, in particular, the health psychological aspects incorporating psychology of learning to foster behavior change. In the next step, the findings were applied to a theoretical framework. For this, we referred to the UTAUT 2 [8,9]. UTAUT 2 encompasses constructs such as (1) performance expectancy, (2) effort expectancy, (3) social influence, (4) facilitating conditions, (5) hedonic motivation, (6) price value, and (7) habit for intention formation as well as the moderating variables age, gender, and experience [8]. Therefore, we recommend the following design suggestions for psychoeducational interventions in AR based on UTAUT 2 for the treatment of CLBP, as exemplified in Figure 2.

### UTAUT 2 Constructs

#### Performance Expectancy

It is recommended to convey psychoeducational content that demonstrates how CLBP can be positively influenced through physical activity [45,46,49,53], emotion management in kinesiophobia [45-49,51], pain-specific coping strategies [45,52,54], and stress and mindfulness techniques [45,49,52-55]. This content can be conveyed through a biofeedback system and supportive agent. As many educational measures are combined with physiotherapy [45-48,52-54], the agent can provide movement exercises, educational content, and interactive stress management techniques [54], supplemented by biofeedback. The biofeedback level and multimodal feedback of avatars promote top-down and bottom-up processes and enable associative learning [50]. To avoid discrepancies between instructions and sensory feedback, facilitate rapid corrections, and enhance user-friendliness [52], the avatar should provide immediate visual-acoustic performance feedback [50]. Biofeedback and body feedback are essential interventions for behavioral modification [17]. It is recommended to combine psychoeducation with a mindfulness-based stress reduction body scan and heart rate biofeedback [51], wherein biofeedback demonstrates progress and enables gamification elements [50,52].

#### Effort Expectancy

Software design should incorporate a gaming environment with in-game level settings to facilitate personalized calibration of movement limitations and body size, particularly for therapeutic activities [51,52]. The system should enable therapists to intervene through a help button or emergency assistance when patients experience pain, anxiety, or perform exercises incorrectly [51]. Frequent feedback for incorrect exercise execution should be avoided, as it may be perceived as disruptive [52].

#### Social Influence

With regard to social influence, an AR intervention should be accompanied by an agent that conveys pain-specific knowledge through lectures as passive knowledge transfer [44] or through support in psychological interactions, such as stress management exercises [54]. An agent can monitor a patient’s body movements to integrate the phenomenon of “virtual body ownership” into the body image or utilize the analgesic effect of the Proteus effect to promote behaviors in the real world [50]. This aligns with BCT‘s recommendations for behavior change, wherein instruction, repetition, and demonstration of behavior have positive effects on physical activity that persist for up to 6 months [50,51]. Therefore, we propose to increase the intention to use by incorporating an agent in an AR intervention, with both passive and interactive roles.

#### Facilitating Conditions

A user-friendly system requires personalized instructions, demonstration videos, and individual briefings. For safety considerations, a spacious environment and wireless head-mounted displays are essential to mitigate the risk of falls. An assistant should be present to support the system. Automated breaks are crucial to prevent overexertion and exacerbation of pain, thereby automatically balancing the active therapy and relaxation periods. The VR headset should be designed for independent removal and application [51].

#### Hedonic Motivation

Passive learning strategies tend to yield superior outcomes in pain and functionality, whereas active methods can elicit significant improvements in knowledge [44]. Gamification demonstrates a motivation-enhancing effect through active and interactive patient engagement [50,52], for instance, through the interactive development of pain-specific knowledge in the Reducept program, where users enter their own brains and shoot with laser guns or connect points [55], or through movement exercises on a simulated farm [52]. A feedback system or biofeedback could be integrated, as outlined in the variable “performance expectancy” of UTAUT 2 and should be phrased positively as praise to increase motivation [51]. Praise as a social reward can occur through interaction with an agent, as described in the variable “social influence” [18]. In CBT, praise serves as positive reinforcement to promote adaptive behaviors and cognitions corresponding to positive CBT, which incorporates positive psychology and solution-focused brief therapy into a cognitive-behavioral context [57]. Gamification in an AR-based intervention enables the implementation of BCTs [17] by creating a material incentive such as within the framework of a game in an AR application [43].

#### Price Value

The headsets required for the interventions should be provided or loaned rather than purchased [50].

#### Habit

Educational programs for CLBP should incorporate a multidisciplinary approach that encompasses both physiological and psychological pain while promoting behavioral modifications such as regular physical activity [49]. Conventional recommendations for behavioral change emphasize repetition as crucial for habit formation [50]. For long-term interventions aimed at behavioral modification, theories addressing the intention-behavior gap and behavioral automaticity in physical activity should be considered, such as the Affective Reflective Theory of Physical Inactivity and Exercise [58] or the Physical Activity Adoption and Maintenance model [59].

### Strengths and Limitations

This scoping review gives an overview of the most important educational content, elements of psychological training, interactive design forms, and relevant pain psychological variables for developing CLBP interventions in AR. It offers a substantiated basis for a theory-based development of a psychoeducational treatment in AR. Thus, this study provides a framework for the theory-driven extraction of hypotheses for future AR research in CLBP treatment. One limitation of this review encompasses the exclusion of certain sport science and physiotherapy databases (eg, SPORTDiscus) and the restriction to studies published in German and English, potentially omitting relevant publications. Further, the distinction between CLBP and chronic nonspecific low back pain in the included studies was often imprecise. The distinction might be relevant for the intervention design, which was neglected in our analysis.

### Recommendations for Research

First, the theoretically proposed design of AR-based psychoeducation for CLBP should be realized in future research. Second, an evaluation of the feasibility and user experience is needed. Third, the therapeutic efficacy of the psychoeducational content in AR must be demonstrated in a clinical evaluation study with patients with CLBP. As no studies on the psychometric properties of measurements in AR are known, psychometric assessments must be tested for measurement equivalence.

### Conclusions

For the development of a framework for an AR-based psychoeducational intervention in CLBP, the combination of theories of acceptance and use of technologies with insights from health psychological behavior change theories appears to be of great importance. An example for a theory-based design of psychoeducation in AR for CLBP is proposed and discussed. Our results offer a substantiated basis for a theory-based development of psychoeducational treatment in AR.

## Appendix

Multimedia Appendix 1 Contents of the education, duration, and physiotherapy involvement.

Multimedia Appendix 2 Summary of the study characteristics, results, and conclusions of the 12 included studies.

Multimedia Appendix 3 Summary of the pain-specific measuring outcomes and instruments.

Multimedia Appendix 4 PRISMA (Preferred Reporting Items for Systematic Reviews and Meta-Analyses) extension for scoping reviews checklist.

## Abbreviations

AR: augmented reality
BCT: behavior change technique
CBT: cognitive behavioral therapy
CLBP: chronic low back pain
PICO: Population, Intervention, Comparison, Outcome
PRISMA: Preferred Reporting Items for Systematic Reviews and Meta-Analyses
UTAUT 2: Unified Theory of Acceptance and Use of Technology 2
VR: virtual reality
