# Supplementary material for: Integration of Conventional and Virtual Reality Approaches in Augmented Reality for Theory-Based Psychoeducational Intervention Design for Chronic Low Back Pain: Scoping Review
Source: Interact J Med Res. 2025 Jan 20;14:e59611. doi: 10.2196/59611 (PMC11791447; doi:10.2196/59611)
Supplement: Multimedia Appendix 1 [file ijmr_v14i1e59611_app1.docx]

**Appendix 1. Contents of the education, duration and physiotherapy involvement**

| **Author** | **Intervention**  **type** | **Including**  **Physiotherapy** | **Duration** | **Contents of the educational intervention** |
| --- | --- | --- | --- | --- |
| Ferlitto  [47] | conventional  Scoping  Review | yes | -- | CBT and CFT interventions: Pain education, pain adaptation strategies, CBT, relaxation tech-niques, cognitive restruckturing, goal reorientation, attention redirection, functional education, coping skills, pain exposure, life-style modification, physiotherapy, manual therapies, mulligan mobilization, joint mobilization, strengthening exercises, motor and sensorimotor control exer-cises, aerobic training, stretch-ing, and physical activity. |
| Rim  [48] | conventional | yes | 3 times a week for 2 hours  (total: 1 month) | Elucidation of spinal anatomy; lumbar plexus pathophysiology via instructional video; move-ment impairment mechanisms; functional adaptation exercises; pain and emotion management; swift mobility restoration; dis-cussion groups for patient ques-tions and concerns, addressing muscle soreness; simplified patient communication during workshops; and a "guide" with key workshop messages, adapted physical activity illustrations, and an educational follow-up program. |
| Sidiq  [49] | conventional | yes | 2 sessions  per week  (total: 6 weeks) | 1.Information on chronic pain,  2. pain neurophysiology,  3. neurobiology,  4. central sensitization,  5. fear avoidance, and  6. social factors influencing  CLBP. The content was  delivered through PowerPoint  presentations, visual aids,  lectures, and interactive  question-and-answer sessions  with the participants. |
| Tomas-Rodriguez [50] | conventional | yes | 5 weeks | Lecture, based on fundamental principles from the book "Explain Pain" by Butler and Moseley (2013):   1. Modification of thoughts, beliefs, and emotions regarding pain experiences 2. Myths, taboos, and existing fears in relation to chronic pain. |
| Janik  [51] | conventional | no | 3 to 5 one-hour sessions  within 15 days | 1.Psychologist:  a. Stress and emotions  management strategies  b.Motivational strategies  (benefits, expierence of exer-  cise, thoughts during PA  2.Kinesiologist:  Principles of PA (challenges,  scheduling, cognitives sup-  port during exercise)  3.Occupational therapist:  One-hour session on manage-  ment of daily PA (Impact of  pain on daily life; positive  impact of regular PA on  CLBP)  4.Social Worker:  Interview on the professional  project (replacement and  occupational retraining) |
| Stamm  [54] | VR | yes | 3 times a week for 30 minutes  (total: 4 weeks) | 1.physiology of pain,  2. pain management,  3. stress management,daily  4. living skills |
| Brown  [55] | VR | yes | 4 sessions with 2 videos  (2.24 to 10.10 minutes) | 1. Educational videos on pain  education:  1.1 Movement is lotion,  1.2 Mindfulness,  1.3 What is pain,  1.4 Hurt Does Not equal  harm,  1.5 Roadmap of changes  2.Guided 360-degree nature videos:  2.1 Ocean,  2.2 Harbor,  2.3 Lake,  2.4 Stream,  2.5. Meadow |
| McConnel [56] | VR | yes | 12 sessions  of  21 minutes each | Lession 1 (On-boarding new patients):  1.1 Intro Pain & Programm, 1.2 Patient Testimonial, 1.3 Rate Current Pain Levels Assessment, 1.4 Perceived Pain Scale, 1.5 Orebro Assessment, 1.6 Nervous System education, 1.7 Practice Deep-Breathing Exercises, 1.8 Conclusion & Expierence Rating  Lession 2 (Understanding the process):  2.1 Rate current Pain Levels Ass-essment, 2.2 Intro to Nervous System, 2.3 Pain Management Strategies Part I, 2.4 Knowledge Retention Quiz, 2.5 Motivation interviews, 2.6 Mindfulness Session  Lession 3 (Introduction to Recovery):  3.1 Rate Current Pain Levels Assessment, 3.2 Global Rating of Change Outcome Assessment, 3.3 Pain Management Strategies Part II, 3.4 CBT: Not Catastro-phizing Pain, 3.5 Pain Catastro-phizing Scale, 3.6 Mindfulness Session, 3.7 Conclusion & Expierence Rating  Lession 4 (Making Better Choices):  4.1 Rate Current Pain Levels Assessment, 4.2 How to Turn Down your Nervous System Sensitivity Part I, 4.3 Mindful-ness Session, 4.4 Conclusion & Expierence Rating  Lession 5 (Overcoming Barrier):  5.1 Rate Current Pain Levels Assessment, 5.2 How To Turn Down Your Nervous System Sensitivity Party II, 5.3 Mindful-ness session, 5.4 Conclusion & Expierence Rating  Lession 6 (Helping Yourself & Others):  6.1 Symptom Custorrized Curri-culum, 6.2 Patient Testimonial Piece, 6.3 Rate Current Pain Levels assessment, 6.4 Global Rating of Change Outcome Assessment, 6.5 Mindfulness session, 6.6 Conclusion & Expierence Rating |
| DeVries [57] | VR | no | Total playing time:  40 - 45 minutes | 1. Phase “Nerves”: visualizing pain perception as a stimulus that could be  targeted with a laser gun, symbolizing control.  Phase 2: “Spinal Cord”: focusing on the visual “pain gates” and breathing to understand the effects of relaxation on pain perception.  3. Brain: Shows how the brain can respond less intensely to pain stimuli by having participants memorize and recreate a pattern of illuminated connection points while focusing on the pain.  4. Alarm Center: The effects of emotions, thoughts and actions on pain sensitivity. The “control room” visualizes the alarm center, which processes pain stimuli and determines their transmission to the brain. |

Note: Co = conventional intervention, VR = virtual reality intervention, PA = Physical Activity
