# Supplementary material for: Integration of Conventional and Virtual Reality Approaches in Augmented Reality for Theory-Based Psychoeducational Intervention Design for Chronic Low Back Pain: Scoping Review
Source: Interact J Med Res. 2025 Jan 20;14:e59611. doi: 10.2196/59611 (PMC11791447; doi:10.2196/59611)
Supplement: Multimedia Appendix 2 [file ijmr_v14i1e59611_app2.docx]

**Appendix 2: Summary of study characteristics, results and conclusions 12 included**

**studies**

|  | **Author** | **Method** | **Results** | **Conclusion** |
| --- | --- | --- | --- | --- |
| 1. | Salazar-Mednez  et al.  (2024) [46] | Chronic pain,  Scoping Review  N = 71 (publication) | The review analyzed 71 articles on the advantages of pain neuroscience education across various instructional techniques and settings. Nonetheless, the programs showed significant variation, often lacking detailed modification descriptions, and the majority failed to consider educational and cultural factors in evaluating the education's effectiveness. | Despite the substantial number of studies on Pain-Neuro-Education and the growing interest in this intervention, there is limited reporting on educational background and cultural aspects in these studies.  Practical implications: It is advised to employ passive and/or active teaching-learning approaches, offered in individual and/or group settings, while taking into account the patient's educational level and cultural background. |
| 2. | Ferlito et al.  (2022) [47] | Systeamtic Review  N =13 | A decadelong literature review identified 13 pertinent articles. Of these, 6 studies showed a significant medium-term symptom reduction. Although only 11 studies assessed disability, 7 reported a notable me-diumterm decrease in the disability index. | Assessing the effectiveness of pain education interventions for CLBP is complicated due to the varied and complex treatments provided to the study participants. Generally, integrating pain education or cognitive behavioral strategies with physical therapy produces superior midterm outcomes compared to physical therapy alone. |
| 3. | Rim et al.  (2022) [48] | CLBP,  Interventiongroup:  Therapeutic patient education + rehabilitation  N = 50  Control group:  Rehabilitation  N = 50 | Evaluations at program start (T0) and end (T1) showed significant improvements in Group A, including reduced pain at rest (P=.00), during work (P=.00), and during physical exertion (P=.03).  Anxiety (P=.03), fear-avoi-dance beliefs (P=.03), kinesiophobia (P=.02) also decreased, with functional capacity improving (P= .00). Depression scores did not significantly change (P=.15). Group B experienced significant reductions in pain at rest (P=.001), during work (P=.03), and during physical activity (P=.00). The cohort showed significant improvements in depress-ion (P=.01), fear-avoidance belief (P=.00), and kinesio-phobia (P=.002). Comparing groups, the combination of TPE and rehabilitation significantly enhanced function (P=.00), anxiety (P= .00), fear-avoidance beliefs (P=.00), and kinesiophobia (P=.00). | Patients with CLBP showed improved functionality and reduced anxiety, misconceptions, and exercise-related fear through a combined educational and rehabilitative approach. |
| 4. | Sidiq et al.  (2024) [49] | CLBP,  intervention group:  N = 46  (physiotherapy + education programm)  Control group:  N = 46  (physiotherapy) | Post-intervention group comparesons indicated that pain education significantly outperformed standard treatment after 6 weeks. There was a marked decrease in disability (mean difference 8.2, P=.001, effect size d = 0.75), a reduction in pain intensity (mean difference 3.5, P=.001, effect size  d = 0.82), and an improvement in the well-being index (mean difference 13.7, P=.001, effect size  d = 0.58). | Incorporating a pain education program enhances the efficacy of conventional physical therapy for CLBP patients. The clinical bene-fits of pain education are evident when combined with standard physical therapy, underscoring its value as a complementary treatment. |
| 5. | Tomas- Rodriguez et al.  (2024) [50] | CLBP,  Education-intervention  Control group:  N =57  Intervention group:  N = 56 | The study assessed 113 participants. Both treatment and control groups showed similar outcomes in pain and fear of movement. At follow-up, the intervention group exhibited lower sensitization and catastrophizing levels, including subscales, compared to the control group. Additionaly, Pain Neuroscience Education led to fewer subjects being classified with central sensitization than in the control group. | Integrating a single Pain Neuroscience Education (PNE) session into the existing back education program did not reduce pain levels but did improve psychological factors such as central sensitization and pain catastrophizing in the medium term. PNE shows promise in improving CLBP management, particularly in time-limited public health contexts. |
| 6. | Janik et al.  (2024) [51] | CLBP,  Education-intervention    Controllgroup:  N= 68  Exerpimtalgroup  N = 68 | At three months, 91% of the experimental group was active, significantly higher than the control group's 77% (P=.001). At six months, the experimental group had a lower attrition rate, with 60% remaining com-pared to the control group's 73% (P=.017). | Incorporating educational com-ponents into care plans enhances patient adherence to treatment and promotes sustained physical activity. Clinical settings should implement educational programs that emphasize motivation, exercise, stress reduction, and daily routines to support individuals with CLBP in maintaining regular physical activity. |
| 7. | Lindner et al.  (2020) [52] | Chronic pain,  narrative review | VR phenomena, such as immersion, presence, embodiment, (virtual) body ownership, and the Proteus effect can alter body perception and behavior. The virtual environment quality, personalized avatars, interaction options, and multisensory inputs enhance immersion and analgesia. Effective VR mechanisms against pain include distraction, cognitive behavioral changes, and reality distance, which lead to neurophysiological cortical changes. Gamification increases motivation, well-being, and adherence to therapy Moderate evidence suggests a positive influence of VR on chronic pain, alt-hough individualized approaches for different pain disorders still need to be investigated. VR can be used as an immersive extension or an alternative to mirror therapy, especially for CRPS or phantom limb pain, with an analgesic effect. |  |
| 8. | Stamm et al.  (2020) [53] | CBP,  VR intervention  Semi-structured interviews  *N* = 10 (patients),  *M_age_*: 75,9, SD = 6.9  + two focus groups:  Physical therapists  (*N*= 3)  Psychotherapists,  (*N*= 2) | The requirement analysis identifies crucial system specifications, including hardware, software, and gamification elements. Essential needs comprise personalized VR exergame applications for specific groups, user-friendly operation, mobility accommodation, daily scenario integration with biofeedback, age-appropriate rewards, and a prescribed 30-minute activity followed by a 15-minute rest. | The identified requirements could potentially be utilized to develop engaging VR exergames that encourage elderly individuals suffering from chronic back pain to engage in consistent exercise routines. |
| 9. | Stamm et al.  (2022) [54] | CBP,  pilot study,  VR-intervention study,  IG: multimodal pain therapy in VR (movement therapy + psychoeducation)  CG: conventional multimodal pain therapy  *N* =22;  *M_age_*: 75.0, *SD* = 5.8  IG: *n* = 11,  CG: *n* = 11 | Two groups of 11 participants were compared, with one receiving multimodal pain therapy using virtual reality (VR) and the other receiving traditional multimodal pain treatment. Both groups experienced a decrease in pain intensity, but the VR group's reduction (MD = 0.64, P=.535) was less than the control group's (MD = 1.64, P=.07). The VR group significantly improved functional capacity from 73.11% to 81.82% (MD = 8.71%; P=.026). Neither group showed significant changes in fear avoidance beliefs or overall physical and mental health. | While the intervention group (IG) did not demonstrate a statistically significant reduction in pain inten-sity compared to the control group (CG), the findings indicated that the current VR application was capable of achieving some level of pain intensity reduction. |
| 10. | Brown et al.  (2023) [55] | CLBP  Prospective,  exploratory study  N = 20 | 15 of the 20 enrolled patients completed the highly rated intervention, but difficulties emerged in implementing the VR headset in a fast-paced clinical setting. Patients' understanding of pain improved for 8 out of 9 key concepts, as evidenced by percentage shifts. | Delivering educational and mind-fulness content via VR headsets to CLBP patients is feasible and well-received. However, integra-ting this technology into fast-paced clinical settings remains challenging due to time constraints, potentially overshadow-ing its benefits. Investigating alternative approaches that mini-mize logistical issues and enhance patient access to content outside clinical environments is crucial. |
| 11. | McConnel  et al.  (2024) [56] | CLBP,  two-arm, parallel, randomized, controlled feasibility study in outpatient clinics  N = 52  Intervention group (VR-Pain Neurscience Education):  n=33  Control group (physiotherapy): n=19 | Out of 595 individuals examined for back pain, 70 had CLBP, 52 participated in the study, and 32 completed it. Adherence rates were 63.6% for VR-Pain-Neuro-Education and 63.2% for standard physiotherapy. VR-Education was found acceptable, with satisfaction scores of 87.37 ± 11.05 compared to 81.17 ± 23.72 for standard physiotherapy. After 6 weeks, no significant differences were found in the measurements of the other outcomes. | The results of the study suggest that VR-PNE may be acceptable and feasible for patients with CLBP. For the next iteration of this study, modifications to study procedures and physical therapy administration should be considered to improve follow-up rates. |
| 12. | DeVries  et al.  (2023) [57] | CLBP,  Education-intervention study  (quantitatively)  Multiple baseline single-case experimental design  N = 8 | Using statistical analyses, this research explores the effects of Reducept, a virtual reality program aimed at improving pain management and educating individuals with CLBP. It provides an indepth examination of individual variations in pain intensity over time. | Additional studies are necessary to explore how Reducept works and its impact on long-term pain disorders. |
