# Supplementary material for: Integration of Conventional and Virtual Reality Approaches in Augmented Reality for Theory-Based Psychoeducational Intervention Design for Chronic Low Back Pain: Scoping Review
Source: Interact J Med Res. 2025 Jan 20;14:e59611. doi: 10.2196/59611 (PMC11791447; doi:10.2196/59611)
Supplement: Multimedia Appendix 3 [file ijmr_v14i1e59611_app3.docx]

**Appendix 3:** Summary of pain-specific measuring outcome and instruments

| **Author** | **Outcome** | **Measurment** |
| --- | --- | --- |
| 1. Salazar-  Medez  [46] | --- | --- |
| 2. Ferltio  [47] | 1. Pain  2. Disability | 1. Nummeric Rating Scale  2. Back pain bothersomeness  3. Visual Analog Scale  4. Brief Pain Inventory  5. Chronic Pain Acceptance  Qustionnaire  6. Roland Disability  Questionnaire  7. modified Roland Disability  Questionnaire  8. Oswestry Disability Index  9. Quebec Pain Disability Score  10. Hannover Functional Ability  Questionnaire |
| 3. Rim  [48] | 1.Pain intensity  (in rest, working,  physical activity)  2. Functional in  lumbago  3. Anxiety, Depression  4. Fear-avoidance-  beliefs  5. Kinesphobia | 1. Pain Visual Analogue Scale  2. Echelle d’Incapacité  Fonctionelle pour l’Evaluation  des Lombalgies  3. Hospital Anxiety and  Depression Scale  4. Fear-avoidance beliefs  questionnaire  5. Tampa Scale of Kinesphobia (TSK-17) |
| 4. Sidiq  [49] | 1. Pain intensity  2. Painspecific  Disability  3. Self-efficacy  4. Well-being | 1. Visual Analogue Scale  2. Roland Morris Disability  Questionnaire  3. General Self-Efficacy Scale  4. Five Well-Being Index |
| 5. Tomas-  Rodriguez  [50] | 1. Pain intensity  2. Identification CS-  Snydrom, comorbid-  dities in chronic pain  3. Kinesphobia  4. Pain catastrophizing | 1. Nummerical pain rating scale  2. Central sensitization inventory  3. Tampa Scale of Kinesphobia  (TSK-11)  4. Pain Catastrophizing Scale |
| 6. Janik  [51] | 1. Pain-related  parameters and  disability  2. Muscle endurance  and flexibility  3. Motivation  4. Physical actvitiy  5. Patient dropout  Note: Physical activity and demolition rate were assessed 3 and 6 months post-treatment. | 1. Annual visal scale  2.Evaluation by a pysican using  a bicycle ergometer  3. Motivation Scale towards  Health-Oriented Physical  Activity  4. Structured interview by a  kinesologist (i.e. including type  of physical activity, intensity,  time, frequency by week)  5. Number of patients |
| 7. Lindner  [52] | --- | --- |
| 8. Stamm  [53] | 1. quality of life  (health status)  2. chronic pain grade  3. interview content | 1. SF-12 Health Survey (German  translation)  2. chronic pain grade  questionnaire  3. semi-structured interviews  main content  categories:  3.1 Part I: Interview with CBP  patients  3.2 Part II: Interview with  physiotherapists and  psychotherapists |
| 9. Stamm  [54] | 1. pain intensity  progression 2. Current pain intensity  3. back pain-related  disability  4. fear-avoidance beliefs 5. General physical and  mental health  6. technology-specific  and psychological  factors (scales:  Curiosity, Anxiety,  Interest, Ease of Use,  Immersion, Use-  fulness, Skepticism,  Accessibility)  7. User Experience | 1. Numeric Rating Scale in a pain  diary 2. Chronic Pain Grade  Questionnaire 3. Hannover Functional Ability  Questionnaire  4. Tampa Scale of Kinesiophobia 5. Health-Survey SF-12  6. Technology Usage Inventory  7. User Experience Questionnaire |
| 10. Brown  [55] | 1. Demographic  variables  2. Feasibility  3. Acceptance  4. Risk stratification  (leg pain, comorbid  pain, disability,  bothersomeness,  catastrophizing, fear,  anxiety, and  depression with a  psychosocial scale)  5. Knowledge and  conviction  (change from initial  knowledge)  6. Patient and therapist  Feedback | 1. investigation of patient records  (Age, gender, education,  medical history, results of  relevant images, information  on current physiotherapy  evaluation and treatment)  2. Dropout rate, Tolerability of  the dose, tolerability of the  training mode, clinical  decision-making by  physiotherapists  3. Recorded experiences of  patients and physiotherapists  with the educational messages,  guided imagery, and VR  training mode  4. Keele StarT back questionnaire  5. Instruments developed by the  authors themselves  6. Positive and negative open-  ended questions |
| 11. McConnel  [56] | 1. Subjective level of  functioning  2. Pain intensity  3. Pain-specific self-  efficacy  4. Pain catastrophizing  5. Patient-perceived  progress  6. Quantification of pain  physiology  7. Fear and avoidance  beliefs  8. Stress recovery  9. Attitudes towards  recovery and negative  circumstances  through CLBP | 1. Oswestry Disability Index  2. Nummeric Pain Rating Scale  3. Pain Self-Efficacy  Questionnaire  4. Pain Catastrophizing Scale  5. Global Rating Scale of Change  6. Neurophysiology of Pain  Questionnaire  7. Fear Avoidance Beleifs  Questionnaire  8. Briefs Resilience Scale  9. Back Beliefs Questionnaire |
| 12. DeVries  [57] | 1. Pain intensity  2. Pain catastrophizing  3. Psychological  complaints  4. Fear of movement  5. Pain coping  6. Quality of life | 1. 11 – point numeric rating scale  2. Pain catastrophizing scale  3. SCL90 – R  4. Tampa Scale for Kinesiophobia  5. Pain Coping Inventory List  6. SF 36 / Rand 36 -item Health  Survey |
